# Complete Genome Sequence of Bacteriophage Deep-Blue Infecting Emetic *Bacillus cereus*

**DOI:** 10.1128/genomeA.00115-16

**Published:** 2016-06-16

**Authors:** Louise Hock, Annika Gillis, Jacques Mahillon

**Affiliations:** Laboratory of Food and Environmental Microbiology, Université Catholique de Louvain, Louvain-la-Neuve, Belgium

## Abstract

The *Bacillus cereus* emetic pathotype is responsible for important food-borne intoxications. Here, we describe the complete genome sequence of bacteriophage Deep-Blue, which is able to infect emetic strains of *B. cereus*. Deep-Blue is a 159-kb myophage of the Bastille-like group within the *Spounavirinae*.

## GENOME ANNOUNCEMENT

*Bacillus cereus* has been implicated in more than 1,000 food-borne intoxications each year in Europe (1), causing either diarrheal or emetic syndromes (2). Bacteriophages represent a promising approach to detect and control pathotypes of *B. cereus*, and consequently improve food safety (3). Here, we report the complete genome of bacteriophage Deep-Blue, a newly isolated myovirus infecting emetic strains of *B. cereus*.

Myovirus Deep-Blue was isolated from an agricultural soil collected in Gembloux (Belgium) through sample enrichment using a mixture of emetic *B. cereus* strains, followed by three single-plaque purification steps using the emetic *Bacillus weihenstephanensis* strain BtB2-4 (4) as host. Morphology was determined using transmission electron microscopy (Mica Technology Platform, UCL). Whole-genome sequencing of Deep-Blue was carried out at Macrogen Inc. (South Korea) using 454 pyrosequencing. Trimmed GS-FLX Titanium reads were assembled in a single contig using the GS De Novo Assembler v2.9 software (454 Life Sciences). The potential coding sequences (CDSs) were predicted using Glimmer v3.02 (5), RAST 2.0 (6), GenMarkS 2.5p (7), and FgenesV (http://www.softberry.com/). All predicted CDSs were functionally annotated using BLASTp searches against the nonredundant NCBI database. tRNAs were predicted using tRNAscan-SE v1.21 (8). EasyFig 2.2.2 (9) and CoreGenes 3.0 (10) were employed to compare the genome of Deep-Blue with other Bastille-like phages at the nucleotide and protein level, respectively.

The Deep-Blue genome spans 158,501 bp with a G+C content of 39.95% and a coding density of 90%. It contains 226 putative CDSs, of which 148 have no predicted functions. The majority of CDSs (192) are transcribed in one orientation. The Deep-Blue genome also contains 19 tRNAs. Predicted CDSs were categorized into seven functional groups: packaging proteins, structural components, proteins implicated in host interaction, phage nucleotide metabolism, DNA synthesis, putative regulatory proteins, and host lysis. Because Deep-Blue also contains type-1 thymidylate synthase (TS1) and dihydrofolate reductase (DHFR) coding genes, it belongs to the recently proposed Bastille-like phage group within the subfamily *Spounavirinae* of the *Myoviridae* (11, 12). Among the 148 hypothetical proteins with no predicted functions, six are unique to Deep-Blue, whereas the others are found in at least one other Bastille-like phage. When the nucleotide sequence of Deep-Blue is compared with the genome of *Myoviridae* phages, a higher synteny is shared with Bastille (13) than with the SPO1 (14) and Twort (15) phages (GenBank accession numbers NC_018856, NC_011421, and NC_007021, respectively). Additionally, a proteome comparison with CoreGenes 3.0 using a BLASTp threshold score of 75 showed that Deep-Blue shares 15% of its proteome with that of Bastille phage, 77% with JBP901 (16), 79% with Bcp1 (17), and 88% with vB_BceM_Bc431v3 (18) (GenBank accession numbers KJ676859, KJ451625, and JX094431, respectively), whereas it shares only 1% with SPO1 and 4% with Twort. Based on these relationships with other Bastille-like phages, the existence of terminal redundancy regions at the genomic ends of Deep-Blue can be expected.

### Nucleotide sequence accession number.

The genome sequence of bacteriophage Deep-Blue was deposited in GenBank under the accession no. KU577463.
